# Effect of Explicit Evaluation on Neural Connectivity Related to Listening to Unfamiliar Music

**DOI:** 10.3389/fnhum.2017.00611

**Published:** 2017-12-19

**Authors:** Chao Liu, Elvira Brattico, Basel Abu-jamous, Carlos S. Pereira, Thomas Jacobsen, Asoke K. Nandi

**Affiliations:** ^1^Department of Electronic and Computer Engineering, Brunel University London, Uxbridge, United Kingdom; ^2^Department of Clinical Medicine, Center for Music in the Brain, Aarhus University & Royal Academy of Music Aarhus/Aalborg, Aarhus, Denmark; ^3^AMI Centre, School of Science, Aalto University, Espoo, Finland; ^4^Experimental Psychology Unit, Helmut Schmidt University, University of Federal Armed Forces, Hamburg, Germany; ^5^The Key Laboratory of Embedded Systems and Service Computing, College of Electronic and Information Engineering, Tongji University, Shanghai, China

**Keywords:** consensus clustering, fMRI, functional connectivity, intentionality, music emotions

## Abstract

People can experience different emotions when listening to music. A growing number of studies have investigated the brain structures and neural connectivities associated with perceived emotions. However, very little is known about the effect of an explicit act of judgment on the neural processing of emotionally-valenced music. In this study, we adopted the novel consensus clustering paradigm, called binarisation of consensus partition matrices (Bi-CoPaM), to study whether and how the conscious aesthetic evaluation of the music would modulate brain connectivity networks related to emotion and reward processing. Participants listened to music under three conditions – one involving a non-evaluative judgment, one involving an explicit evaluative aesthetic judgment, and one involving no judgment at all (passive listening only). During non-evaluative attentive listening we obtained auditory-limbic connectivity whereas when participants were asked to decide explicitly whether they liked or disliked the music excerpt, only two clusters of intercommunicating brain regions were found: one including areas related to auditory processing and action observation, and the other comprising higher-order structures involved with visual processing. Results indicate that explicit evaluative judgment has an impact on the neural auditory-limbic connectivity during affective processing of music.

## Introduction

Although the enjoyment of music is a very common phenomenon, it is not always the result of an explicit choice, since music often accompanies daily activities such as shopping or TV watching. According to a study by Sloboda and O'Neill (2001) using the experience sampling method, about 44% of the events recorded involved music but in only 2% of them music was listened to attentively. In these instances of casual and unfocused listening, we do not necessarily carry out a conscious evaluation of the music heard in terms of aesthetic properties, such as beauty, structure or mastery. According to music psychologist Sloboda (2010), in everyday life the expression and induction of basic emotions such as joy or sadness by music are prioritized over “aesthetically tinged” emotions such as deep enjoyment, awe or frissons (Sloboda, 2010, p. 503). According to a recent account (Nieminen et al., 2011; Brattico and Pearce, 2013; Brattico et al., 2013; Brattico, 2015; Reybrouck and Brattico, 2015), a full musical experience includes final outcomes such as aesthetic emotions (e.g., enjoyment or pleasure, often accompanied by bodily changes such as goose bumps on the skin, accelerated heartbeat, or tears in the eyes), aesthetic judgments (“this music is so beautiful”), and the formation of specific preferences and musical taste (“I love chamber music”). In a broader framework encompassing all experiences of an art object, Chatterjee and Vartanian (2016) propose that all art phenomena emerge from the interaction between three main mental and neural systems, a sensory-motor one (sensation, perception, motor system), a knowledge-meaning one (expertise, context, culture), and an emotion-evaluation one (reward, emotion, wanting/liking). Also in the framework by Juslin (2013) aesthetic judgment was viewed as the final outcome of several different emotion-induced mechanisms. On several accounts, though, additional factors are listed that enable the listener to reach a full aesthetic experience. One that is considered especially crucial is a dedicated, decisional act of judgment toward the art object (Brattico and Pearce, 2013; Brattico et al., 2013; Hodges, 2016). Even if this factor is feasible to study, very little research has been dedicated to determine its role in an aesthetic response during music listening, such as pleasure or enjoyment.

In an analytic study of aesthetic processes in the visual modality, Höfel and Jacobsen (2007a) instructed participants to view passively abstract black and white patterns or to contemplate them aesthetically, i.e., to reflect upon the beauty of those shapes, although without giving an overt aesthetic judgment. The electric brain potentials elicited during the two experimental conditions evidenced that evaluative processes occurred during attentive contemplation only and not during mere viewing, as indexed by a late positive potential visible only in the contemplation condition. Furthermore, neither conditions elicited the early frontocentral negativity to “not beautiful” shapes (reflecting impression formation, namely the first integrated representation of the stimuli derived from the sensory information provided). This finding was different from what was previously observed during tasks involving overt aesthetic judgments (Jacobsen and Höfel, 2003). Hence, the authors postulated a distinction between aesthetic mode or “central processes of thinking about aesthetic value” and “deciding upon an aesthetic judgment.”

In terms of brain structures distinguishing explicitly from involuntary pleasure, a rare meta-analysis by Kühn and Gallinat (2012) has combined results from 39 neuroimaging studies related to pleasure as induced by odor, taste, music, or visual stimuli. Overall, positive correlates of conscious, subjective pleasure were selectively obtained in medial orbitofrontal cortex, left nucleus accumbens (ventral striatum), pregenual cortex, left thalamus, and mid cingulate cortex. Several of those structures are consistently found in relation to motivational stimuli, as well as expected or reward anticipation (Mueller et al., 2015). Particularly, the nucleus accumbens is described as the “hedonic hot spot” of the brain (Peciña et al., 2006). These results replicated a previous meta-analysis by Brown et al. (2011). The latter study additionally identified the anterior insula as a hub common to all sensory modalities in association with pleasurable stimuli (whether consciously evaluated for their affective qualities or not). In the meta-analysis by Kühn and Gallinat (2012) the clusters of activation found in the selected studies were further subdivided into the ones in which participants judged pleasantness during scanning (18 studies) from those in which they judged the stimuli outside the scanner (11 studies). The main interest by the authors of the study was in testing the hypothesis of a medial orbitofrontal function for self-referential processes involving conscious hedonic decisions. However, no difference was found, although a relation between left amygdala activation and making conscious pleasure judgments during scanning was noticed. This meta-analysis, while commendable in trying to discern neural correlates of distinct psychological processes, puts forward the need for further empirical work within each sensory modality.

In the music domain, a growing number of studies (including some meta-analyses) has looked at the brain structures and neural connections associated with perceived or felt musical emotions. Nevertheless, very little knowledge has been accumulated on explicit judgments during these emotional experiences. A rare attempt to study recognition of affect with neuroimaging has been done by Bogert et al. (2016). In their study 30 music excerpts, each lasting 4 s, from blockbuster film soundtracks were presented twice to subjects in two separate (counterbalanced) conditions. In one condition, subjects were asked to pay attention to the numbers of instruments playing in the clip (implicit condition). In the other condition, they were instructed to explicitly classify the emotions conveyed by the music (explicit condition). In the implicit condition (contrasted with the explicit one) the music stimuli activated bilaterally the inferior parietal lobule, premotor cortex, as well as reward-related areas such as the caudate (dorsal striatum) and ventromedial frontal cortex. In contrast, dorsomedial prefrontal and occipital areas, previously associated to emotion recognition and cognitive processing of music, were active during explicit judgment of musical emotions. Indeed, according to the conceptual-act model of emotions by Barrett and Wager (2006) and Lindquist et al. (2012), discrete emotions occur only after the neurophysiological states of valence and arousal (which form what is called “core affect”) meet with an act of categorization and labeling happening in dorsolateral prefrontal and parietal cortices. This conceptual act occurs “in the moment” and uses pre-existing knowledge of emotions and language systems in the brain to attribute a lexical category (Barrett, 2006).

In a recent experiment, the chronometry of the neural responses during categorization of musical emotions by using neurophysiological methods was studied (Ellison and Brattico, 2015). They chose a very simplified paradigm in order to measure the phase-locked event-related responses allowing very fine temporal resolution of the order of milliseconds. Stimuli were chord cadences, ending with a major or minor chord that could be tuned or mistuned in the middle note. Results showed that negatively rated (sad or incorrect) cadence endings in both tasks elicited early neural responses whereas only later responses, peaking at around 500 ms, differed between sad and incorrect stimuli. This suggests a neural chronometry of music listening in which feature encoding and sensory memory processes are followed at a medium latency by affective classification, after which an evaluative stage takes place (similar findings have been obtained also in the visual domain (Jacobsen and Höfel, 2003; Höfel and Jacobsen, 2007b; Jacobsen, 2014). The experiments by Bogert et al. (2016) and Ellison and Brattico (2015), although clarifying the influence of explicit classification on the neural processing of discrete emotions in music, did not address how the enjoyment of music is affected by simultaneously listening to music and making an explicit judgment of the same music.

Furthermore, these previous efforts within the field of aesthetic music research had ignored an important property of the human brain, namely its intrinsic connectivity and constant inter-communication between its citoarchitectonically and functionally different regions (van den Heuvel et al., 2008; Wilkins et al., 2014). The interest toward this crucial brain property has grown immensely in the recent years, leading to the formation of a novel discipline termed network neuroscience (Bassett and Sporns, 2017). To account for affective processes and particularly pleasure (both from primary activities and from art and music), the earlier notions of simple self-stimulating pleasure centers deep in the brain have been abandoned in favor of an accepted view of a pleasure system relying on the “balanced interaction over time of key brain regions” (Kringelbach and Berridge, 2017). Hence, the study of this interaction between neural networks is increasingly carried out using fMRI and functional connectivity, namely the interdependency of hemodynamic fluctuations (related to neural activity) in the BOLD time-series, which can be interpreted as a measure of communication between brain regions (Hutchison et al., 2013; Alluri et al., 2017; Betzel et al., 2017). Functional connectivity is nowadays considered central for answering the question of how form constrains function (Bassett and Sporns, 2017). Within the music domain, to our knowledge, only three studies have thus far looked at functional connectivity in relation to liking of songs and preference for musical genres (Salimpoor et al., 2013; Wilkins et al., 2014; Alluri et al., 2015). However, these studies examined the BOLD changes either only during free listening of music that varied in familiarity, or only during focused evaluation without discerning the role of explicit evaluative vs. spontaneous affective processes during focused listening.

In the majority of previous studies the focus has been on regional activity rather than connectivity between brain structures. In the present study, we aimed to complement the limited available literature, in which mainly attentive paradigms have been used (e.g., Salimpoor et al., 2013; Wilkins et al., 2014) or regional brain activity to musical enjoyment have been investigated (Pereira et al., 2011). To achieve this goal, we set out to depict whether explicit liking judgments of music, as opposed to descriptive judgments of the music or to listening *per se*, are necessary to co-activate limbic (e.g., thalamus, hippocampus, parahippocampal gyrus, amygdala, hypothalamus) and reward (nucleus accumbens, orbitofrontal cortex, insula, ventromedial prefrontal cortex) regions of the brain. In other words, our first aim is to determine whether enjoyment is a spontaneous affective process that can occur during concentrated listening, even without requiring an explicit decision and even when attention is diverted toward some specific aspects of the music. Moreover, as the complementary aim we set out to isolate the neural circuits specifically recruited during conscious aesthetic evaluation of music.

To these aims, we measured healthy adult volunteers with fMRI while they passively listened to 15 s music excerpts selected by the experimenters, varying in musical genre, acoustic features, and emotional connotations, as well as while they listened and classified the excerpts based on the gender of the singer or based on whether they enjoyed the excerpts or not. We decided to focus on studying the neural connections during aesthetic listening of music, based on the shared assumption that the complex physiological activity of aesthetic-related neural systems is determined by the patterns of connections between their elements rather than by a one-to-one mapping between a single region and a function (Lindquist et al., 2012; Bassett and Sporns, 2017; Pelowski et al., 2017). In summary, we set the aim to inspect how functional connectivity changes across the entire brain during the aforementioned three experimental conditions (naturalistic listening/liking judgment /gender judgment). For the analysis of the interconnected neural networks, we adopted the binarisation of consensus partition matrices (Bi-CoPaM), a tunable consensus clustering paradigm that combines the results from various clustering methods to identify the subset of voxels that are consistently correlated under different circumstances (Abu-Jamous et al., 2013a, 2015). Adapted to fMRI data, the Bi-CoPaM is able to find brain structures that consistently have very similar intrinsic temporal patterns of coherent neural activity during certain experimental conditions. In our previous work (Liu et al., 2017), this approach successfully identified the brain structures functionally connected during evaluative liking judgments of familiar music as opposed to emotional judgments. That study did not address whether the focus on an aesthetic evaluative judgment (such as conscious liking) would drive the functional connectivity, especially of the reward and attentional brain networks, or whether this connectivity would be triggered irrespectively of the listening goal. In this study, we predicted co-activation in a network of mesiotemporal limbic structures, including the nucleus accumbens, in response to the liked musical stimuli. This is irrespective of the experimental task performed by the subjects, namely irrespectively of whether they were focusing on making a liking evaluation or not. In turn, we anticipated functional connectivity within prefrontal and parietooccipital regions specifically in association with the conscious decision processes of liking judgment. Moreover, we predicted that the decision process might down-regulate the activity and connectivity of the limbic and reward networks during listening to the liked music excerpts. For the disliked musical excerpts, we expected the recruitment of the amygdala, insula and auditory cortices, similarly to our previous study including liked and dislike music across all the experimental conditions, but particularly for the conditions not requiring the conscious liking decision (Burunat et al., 2015).

## Materials and methods

### Participants

Twenty-five healthy volunteers (16 females and 9 males) without any hearing, neurological or psychological problems were included in this study. The experiment was approved by the ethical committee of the coordinating Uusimaa and Helsinki Hospital and complied with the Helsinki Declaration. Written informed consent was obtained from every participant in this study.

### Stimulation

The experimenters selected 36 audio excerpts with English lyrics, lasting 15 s each, taken from commercially available pop/rock songs (familiar to all Finnish subjects as they all learn English since primary school). Although all excerpts belonged to pop/rock genres, they varied in their subgenre, ranging from mainstream soft pop to heavy metal and indie rock. This variation in subgenres was made to allow for a wide range of acoustic features and emotional connotations that would minimize the possible confound of brain effects in response to specific sensory or emotional aspects of the stimuli, as well as to obtain contrasting musical preferences between participants. For instance, we expected that participants favoring indie rock would dislike mainstream pop and vice versa. Please see supplementary data sheet 1 for a complete list of stimuli used in this study.

### Procedure

The experiment was conducted at the Advanced Magnetic Imaging (AMI) Center at Aalto University, Espoo, Finland. Before the experiment, participants were contacted by e-mail and asked to name three or four genres (or sub-genres) of music that they prefer and other three or four genres that they strongly dislike, along with examples. This information was used to select songs within three different sub-genres of the pop-rock repertoire that could accommodate the preferences of all the participants, as described in Section Materials and Methods Stimulation. Upon arrival at the laboratory, before entering the scanner, participants were asked to listen to the stimuli to allow them to be equally familiar with them and thereby to minimize the possible bewilderment from unfamiliarity factor, which was previously shown to have a strong influence in the pleasure response of listeners (Pereira et al., 2011). Subsequently, they changed their clothes and were prepared to enter the scanner room. Participants' fMRI responses were acquired while they listened to each of the musical stimuli in random order yet the order was unique to each participant as well as in each listening condition. The stimuli were delivered to participants via high-quality MR-compatible insert earphones (Klaus A. Riederer, KAR, ADU2a) and plastic tubes. The sound level of the stimuli was adjusted for each subject so that the stimuli were audible above the scanner noise, but the volume stayed within safety limits (below 80 dB). Additional hearing protection was used (conventional noise-attenuating headphones). Moreover, the scanner noise was attenuated with foam cushions placed at the side of participant's head. For more details about the sound apparatus used at AMI Center, see http://ani.aalto.fi/en/ami_centre/facilities/stimulus_systems/auditory_system/.

In the scanner, participants' tasks were the following, presented in subsequent blocks prompted by text presented on the screen:

1. Naturalistic listening block: participants were asked to listen to each music stimulus naturally, without making any explicit judgments, and to press one out of two buttons after each stimulus.
2. Descriptive Gender judgment block: participants were asked to determine the gender of the singer in each music excerpts presented by pressing one out of two buttons.
3. Evaluative Liking judgment block: participants were asked to decide by pressing one out of two buttons whether they like or dislike each excerpt.

**Figure 1:**
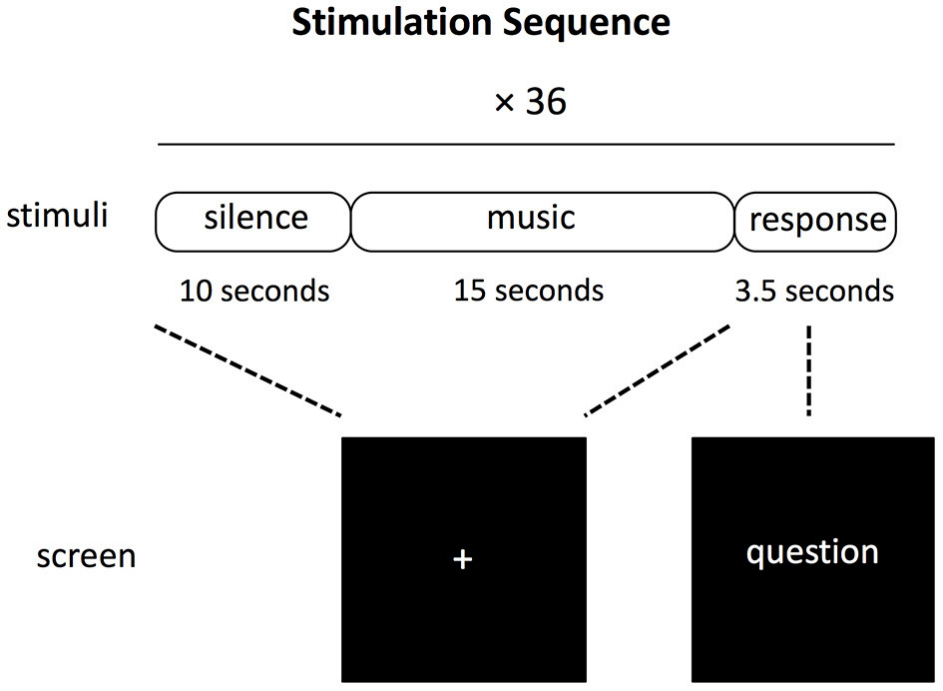
Schematic representation of the experimental trial used for each of the three blocks constituting the experiment (naturalistic listening, gender judgment, and liking judgment).

The first block of the experimental session was always the naturalistic listening because one arguably cannot go back to the naturalistic listening state after having performed one of the other two judgment tasks. The gender judgment block and liking judgment block were counterbalanced across subjects. During each music excerpt, the participants were asked to look at the fixed cross symbol at the center of screen. After each 15-s long music excerpt, there was a 3.5 s interval for the participants to response to the question shown on the screen by pressing either a button joystick that was kept in the left hand or a button joystick that was kept in the right hand. The order of the response buttons (e.g., left for like, right for dislike) was counterbalanced between subjects and indicated by the position of the words in the screen for the two explicit judgment tasks. For the naturalistic task, the participants were asked to press alternatively each button. After each trial, there was a 10 s long period before the next music excerpt was presented. Figure 1 illustrates the experimental protocol in each block.

### fMRI data acquisition and preprocessing

Scanning was performed using a 3T MAGNETOM Skyra whole-body scanner (Siemens Healthcare, Erlangen, Germany) and a standard 20-channel head-neck coil, at the AMI Centre. Using a single-shot gradient echo planar imaging (EPI) sequence 33 oblique slices (field of view = 192 × 192 mm; 64 × 64 matrix; slice thickness = 4 mm, interslice skip = 0 mm; echo time = 32 ms; flip angle = 75°) were acquired every 2 s, covering the whole-brain for each participant. T1-weighted structural images (176 slices; field of view = 256 × 256 mm; matrix = 256 × 256; slice thickness = 1 mm; interslice skip = 0 mm; pulse sequence = MPRAGE) were also collected for individual co-registration.

Functional MRI scans were preprocessed on a MATLAB platform using SPM8 (Statistical Parametric Mapping), VBM for SPM (Voxel Based Morphometry; Wellcome Department of Imaging Neuroscience, London, UK), and customized scripts developed by the present authors. For each participant, low-resolution images were realigned on six dimensions using rigid body transformations (translation and rotation corrections did not exceed 2 mm and 2° respectively), segmented into gray matter, white matter, and cerebrospinal fluid by using VBM, and registered to the corresponding segmented high-resolution T1-weighted structural images. These were in turn segmented, realigned, and spatially normalized to the MNI (Montreal Neurological Institute, Evans et al., 1994) templates using a 12-parameter affine transformation. Functional images were then smoothed to best accommodate anatomical and functional variations across participants as well as to enhance the signal-to-noise by means of spatial smoothing using 8 mm full-width-at-half-maximum Gaussian filter.

For preparing the data for the consensus analysis we used the fMRItoolbox (implemented at the University of Jyväskylä in MATLAB environment, e.g., used in Alluri et al., 2012; Burunat et al., 2015, 2016). Firstly, the 3D volume data was converted to a vector (228453 × 1) by using a standard brain mask. The above step had been applied to every 3D volume scan from each participant and all the scans were combined sequentially, forming the fMRI time series of each individual. The time series were high pass filtered with a cutoff frequency of 1/120 Hz to remove the linear trend and scanner drift. Then, for each participant, according to the order that musical excerpts were played, the whole fMRI time series were segmented into 36 EPI brain volumes, each containing 7 or 8 time points (covering 15 s at a sampling rate of 2 s), and each corresponding to instances when the participants were listening to music clips. For each participant, all the 36 music excerpts were labeled as liked or disliked according to the responses the participants gave to each music excerpt in liking judgment block. In gender judgment block and naturalistic listening block, although the participant did not perform the liking judgment, the excerpts were still labeled as liked or disliked according to the response each excerpt receive in liking judgment block. This was used to study the effect of listening to liked or disliked music when participants were not actively performing liking judgments. In summary, there were six categories of music excerpts from each participant:

1) LL: in liking judgment block music excerpt is liked(19 excerpts avg. STD 7.3)
2) LD: in liking judgment block music excerpt is disliked (17 excerpts avg. STD 7.3)
3) GL: in gender judgment block music excerpt is liked (19 excerpts avg. STD 7.3)
4) GD: in gender judgment block music excerpt is disliked (17 excerpts avg. STD 7.3)
5) NL: in naturalistic listening block music excerpt is liked (19 excerpts avg. STD 7.3)
6) ND: in naturalistic listening block music excerpt is disliked (17 excerpts avg. STD 7.3)

For each category, a different participant might have a different number of music excerpts. In total, the average number of liked and disliked music per participant are roughly the same (liked: 19 excerpts, disliked: 17 excerpts).

### Consensus clustering methods

For this study, we used the Bi-CoPaM tunable consensus clustering paradigm (See supplementary data sheet 1) to identify the brain structures consistently functionally connected during processing of liked or disliked music during the aforementioned three experimental conditions. The following briefly explains the procedures of applying Bi-CoPaM. Firstly, individual partition (clustering result) is generated for the same set of fMRI time series by using one clustering algorithm on a selected dataset. By applying C different clustering methods to L different datasets measuring the BOLD responses from one type of stimulus, R (= C × L) partitions are generated. Then these R partitions are aligned and averaged, forming a fuzzy consensus partition matrix (CoPaM) where each entry in each cluster represents the fuzzy membership value based on the number of individual partitions assigned to it. Lastly, the fuzzy CoPaM is binarised to obtain a binary consensus partition matrix containing the final membership for each voxel. One important feature of the Bi-CoPaM is that the results are tunable in terms of the level of correlation within each cluster by setting parameter δ to control the tightness of the final clusters. The readers are referred to Abu-Jamous et al. (2013a) and Liu et al. (2017) for more technical details. For a brief introduction regarding the key steps in the consensus clustering method, please see supplementary data sheet 1.

### Clustering experiment

#### Individual clustering result generation

Each excerpts data (normalized to zero mean and unit variance) was clustered by K-means (Chuang et al., 1999; Kahnt et al., 2012), hierarchical clustering (Ferrarini et al., 2009; Blumensath et al., 2013), and self organizing maps (SOM) (Peltier et al., 2003; Liao et al., 2008) with *K*-values of 10, 25, 50, and 100 respectively. The clustering index for each excerpt data had a label that was the same as the label of the corresponding music excerpt (LL, LD, GL, GD, NL, and ND). These labels would be used for differentiating different datasets in the following consensus clustering analysis.

The corresponding datasets were fed into Bi-CoPaM analysis paradigm to identify the brain areas that responded consistently and similarly in the following conditions,. For instance, by combining the clustering results from datasets LL and LD, the brain structures that consistently showing synchronized BOLD responses during liking judgment tasks were identified.

1) Liking judgment block (LL, LD)
2) Gender judgment block (GL, GD)
3) Naturalistic listening block (NL, ND)
4) All the liked music excerpts (LL, GL, NL)
5) All the disliked music excerpts (LD, GD, ND)

#### Cluster filtering

After the clusters were generated by Bi-CoPaM, the following filterings were applied. Firstly, the initial clusters from the Bi-CoPaM were filtered by hypergeometric distribution test to exclude possible randomly included structures within each cluster. Voxels within a certain brain structure covering large connected brain area would feature a very small *p*-value (normally below 0.001 level) while those covering tiny scattered brain structures would result in a relatively high p value (normally above 0.1 level). We chose p equals to 0.001 to distinguish the major brain structures from the minor brain structures within each cluster. Secondly, the original clusters were filtered by discarding those voxels with weak responses (voxels whose time series have a very small variance), since the data used to be clustered were normalized and thus lost the signal magnitude information. In this analysis, the criteria of keeping a voxel is that its variance has to be greater than half of the mean of the variance for all the voxels in a particular cluster. Then if more than 70% of the subjects showed a strong response at a certain voxel, this voxel was retained. Finally, we used the fMRItoolbox developed by University of Jyväskylä to remove the scattered tiny clusters. The reason we chose these thresholds is to keep those voxels having big fluctuations in clusters. Obviously a higher threshold produces more conservative results (and the smaller the clusters are). We do not claim these thresholds are absolutely optimal. It is rather an exploration of the novel analysis strategy. It has been shown in our previous study (Liu et al., 2017) that these parameters do not distort the results much, on the contrary, they are meant to keep the large continuous voxels showing relatively strong BOLD activities.

### Comparing the clustering results among different listening conditions (topological interaction)

After the consensus clustering results were obtained for each of the three listening conditions (liking judgment, gender judgment, naturalistic listening), we used the set operations to compare the differences of the clustering solutions between any two listening conditions, which is called cluster topological interaction. Assume there are two clusters A and B, we denote the common voxels between A and B as A ∩ B, The exclusive voxels for A is (A − A ∩ B), and the exclusive voxels for B is (B − A ∩ B). For more complex relationships, e.g., (A − A∩ B − A ∩ C), the interpretation follows in the same manner. In the results section, when the topological interaction between clusters are demonstrated, these annotations will be used. These set operation symbols will make the comparison between two clustering solutions more concise, where each clustering solution might consist of more than one cluster.

## Results

In experimental condition, we inspected the first 20 clusters (ranked by M-N plots algorithm used in Abu-Jamous et al., 2013b, 2014a, 2015), as these clusters showed very strong similarity in the response shapes as well as covered large continuous regions, which complies with expectations based on knowledge of brain physiology. After ordered selections of clusters from M-N scatter plot, all scattered voxels were removed. Subsequently, only those clusters still covering large continuous regions were kept and investigated in the following.

### Consensus clustering results

The topology of clusters in each experimental condition is rendered on a standard structural 3D brain. Each cluster has a number (e.g., C3), indicating its order selected by the MN-plot technique, and marked with a color to be distinguished from the other clusters. The set of clusters in each of the following three studies have been obtained separately. Within a study, the top ranked cluster (by the MN plot) is labeled C1 and the fifth ranked cluster is labeled C5. It should be noted that C5 in liking judgment, C5 in gender judgment, and C5 in naturalistic listening have been independently obtained and each of them is an independent cluster.

#### Liking judgment

**Figure 2:**
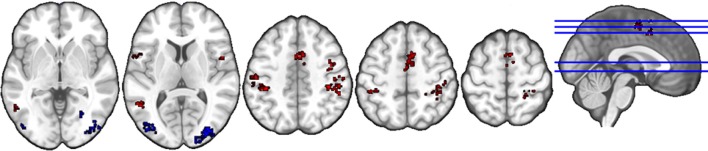
Topology of clusters from liking judgment condition. Red area is C3 and blue area is C5.

The anatomical labels, size, and MNI coordinates are put into Supplementary Table 1. Figure 2 illustrates the topology of clusters from liking judgment. Cluster C3 (red) comprises areas such as the supramarginal and postcentral gyri, possibly related to language and somatosensory processing as well as the middle temporal gyrus, Rolandic operculum, and inferior frontal gyrus, previously associated with the cognitive processing of sounds. In addition, brain regions related to action observation and motor preparation, such as the supplementary motor area (SMA), the precentral gyrus, are also included, as well as the bilateral angular gyri. Cluster C5 (blue) mainly includes higher-order structures involved with visual information processing, namely the cuneus, lingual gyrus, middle, inferior and superior occipital gyri, and fusiform gyrus.

#### Gender judgment

**Figure 3:**
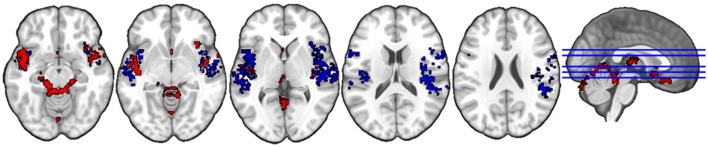
Topology of clusters from gender judgment condition. Red area is C3 and Blue area is C5.

The anatomical labels, size, and MNI coordinates are put into Supplementary Table 2. Figure 3 illustrates the topology of clusters from gender judgment. Cluster C3 (red) includes three major systems, namely the auditory processing system (middle and superior temporal gyri) limbic system (thalamus, amygdala, parahippocampal gyrus, orbitofrontal gyrus, insula, putamen) and the cerebellum. Cluster C5 (blue) comprises a broad area of the auditory cortex and the bilateral insula. Other structures interconnected in this cluster are the bilateral inferior frontal gyrus, the SMA, the right supramarginal gyrus, the precentral and postcentral gyri, plus a small bit of the right putamen.

#### Naturalistic listening

**Figure 4:**
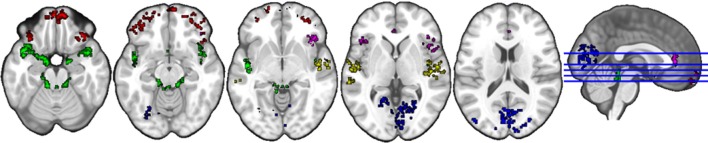
Topology of clusters from naturalistic listening condition. Red area is C4, blue area is C5, green area is C6, violet area is C8, yellow area is C9, and cyan area is C11.

The anatomical labels, size, and MNI coordinates are put into Supplementary Table 3. Figure 4 illustrates the topology of clusters from naturalistic listening. Cluster C4 (red) includes various parts of the orbital frontal cortex (inferior, middle, and superior) extending to a small part of the left middle and inferior temporal gyrus. Cluster C5 (blue) mainly contains structures related to visual processing, e.g., occipital gyrus, cuneus, and fusiform gyrus. Cluster 6 (green) comprises structures that have a similar topology to the cluster C3 in gender judgment condition, namely the auditory and limbic system plus a small part of cerebellum. Cluster C8 (violet) includes bilateral anterior cingulate and paracingulate gyrus as well as bilateral insula, where the left insula is very small compared to the right part, plus various positions of inferior frontal cortex (triangular, orbital, and opercular). Cluster C9 (yellow) is a combination of the auditory processing related structures (middle and superior gyrus, Heschl's gyrus) and right insula. Cluster C11 (cyan) is the smallest one including three structures within the right hemisphere.

### Cluster topology interaction

We compared the differences in clusters from two experimental conditions using a Venn diagram to show the overlaps and exclusive brain structures between any two sets of clusters from corresponding experimental conditions. For example, “C5_L − C5_N ∩ C5_L” represents those voxels only belonging to Cluster 5 in liking judgment condition and not found in Cluster 5 of the naturalistic listening condition. In Supplementary Tables 4–6 the anatomical information of overlapped structures and exclusive structures are extracted, enabling the inspection of the topology relationships between two sets of clusters.

**Figure 5:**
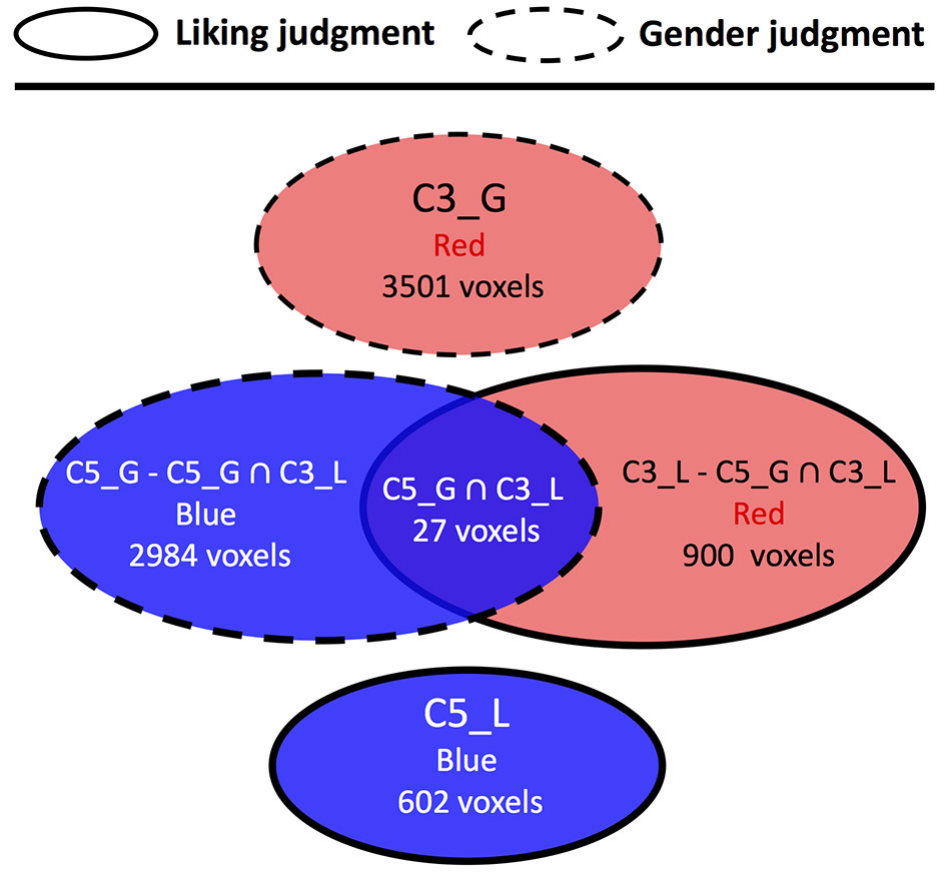
Cluster topology interaction between liking judgment and gender judgment. Circles representing clusters from liking judgment condition have solid border and circles representing clusters from gender judgment condition have dashed border. The affix after each cluster number also reflects which listening condition this cluster belongs to. G means gender judgment session, and L means liking judgment session The color label within each circle indicates the color of the corresponding cluster, and the number of voxels indicates the size of different clusters/brain regions.

**Figure 6:**
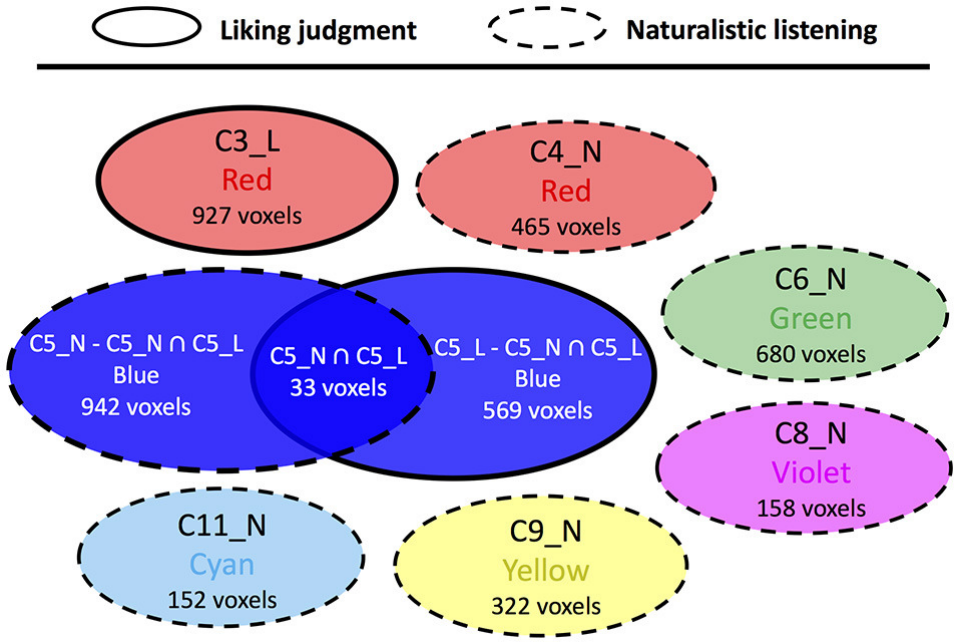
Cluster topology interaction between liking judgment and naturalistic listening. Circles representing clusters from liking judgment condition have solid border and circles representing clusters from naturalistic judgment condition have dashed border. The affix L means a certain cluster is from liking judgment session and N means naturalistic listening session.

For the comparison between liking judgment and gender judgment conditions (Figure 5, Supplementary Table 4), only one overlapped area in C5 was found. The structures included are scattered across various brain regions such as postcentral gyrus, inferior frontal cortex, supramarginal gyrus, precentral gyrus, and other two other structures having only one voxel each. The comparison between liking judgment and naturalistic listening (Figure 6, Supplementary Table 5) showed almost no overlapping between the two sets of clusters. Only 27 voxels belonging to the visual sensory system are shared by C3 in liking judgment condition and C5 in gender judgment condition.

**Figure 7:**
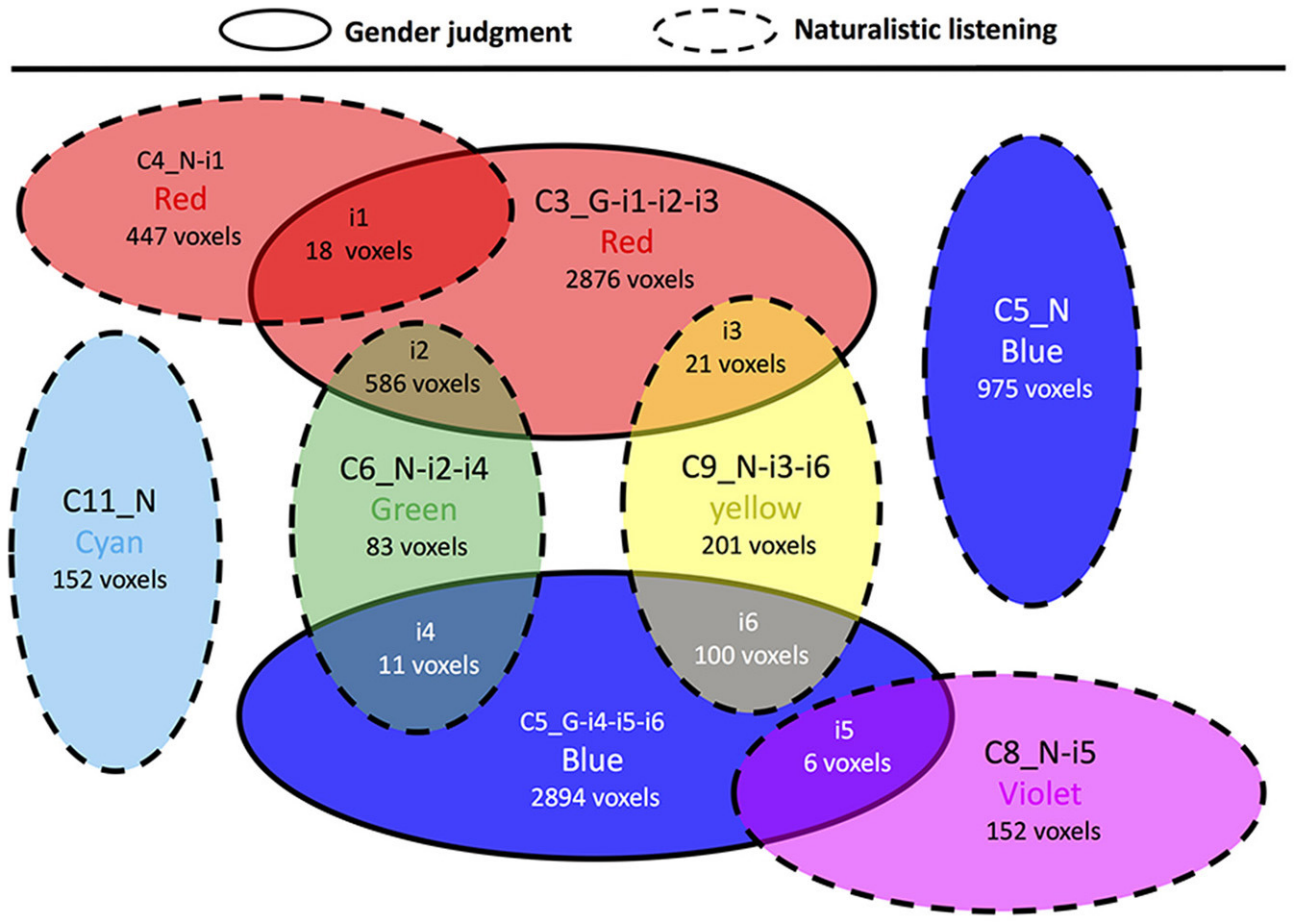
Cluster topology interaction between gender judgment and naturalistic listening. Circles representing clusters from gender judgment condition have solid border and circles representing clusters from naturalistic judgment condition have dashed border. The affix G means a certain cluster is from liking judgment session and N means naturalistic listening session.

In contrast to the previous comparisons, the cluster topology interaction between gender judgment and naturalistic listening showed several overlapping areas (Figure 7, Supplementary Table 6). The two clusters in gender judgment (C3 and C5) overlaps with four out of six clusters in naturalistic listening (C4, C6, C8, and C9). Among all the overlapped areas (i1 to i6), i2 and i6 contain large numbers of voxels. Area i2 is the intersection between the auditory-limbic systems in gender judgment and naturalistic listening respectively, plus the difference in cerebellum. Area i6 contains the overlapped auditory cortex between the two experimental conditions. Other overlapped areas (i1, i3, i4, and i5) are smaller and contain no more than two structures as shown in Supplementary Table 6.

## Discussion

In this study, we applied the novel tunable consensus clustering paradigm Bi-CoPaM to study whether and how the conscious evaluation of the music heard in terms of aesthetic properties would modulate emotion-, reward-, and attention-related brain connectivity. By using this novel clustering approach, we obtained distinct neural networks subserving music enjoyment during three levels of engagement with the music. These refer to naturalistic listening, not involving an overt liking judgment, to non-evaluative judgmental listening involving an explicit descriptive (non-evaluative) judgment task up to conscious listening aimed at issuing an explicit evaluative liking judgment of the music. Results support our hypothesis on the impact of evaluative judgments on brain-networks related to music enjoyment, with the down-regulation of auditory-limbic connectivity during conscious liking judgments and the increased connections between audio-motor and attention-related regions. More specifically, the obtained clusters clearly point to auditory-limbic connectivity between areas, such as thalamus, superior temporal gyrus, amygdala, and parahippocampal gyrus, or between orbitofrontal regions or between supratemporal regions, insula and putamen. This was observed only during non-evaluative (but attentive) listening, namely when participants were asked to either classify the gender of the voice in the music excerpts or to simply passively listen to them. When participants were asked to decide whether they liked or disliked the music excerpt, only two clusters of intercommunicating brain regions were found. One includes regions related to cognitive processing of sounds (middle temporal gyrus, rolandic operculum, inferior frontal gyrus), and regions related to action observation and motor preparation (supplementary motor areas, precentral gyrus). The other comprises higher-order structures involved with visual processing (cuneus, lingual gyrus, middle, inferior and superior occipital gyri, fusiform gyrus).

The choice of the novel consensus clustering paradigm is an answer to the recent criticisms of the most common analysis methods in fMRI (Eklund et al., 2016). Typically, an fMRI study on a certain stimulation paradigm would adopt a single method of analysis and statistical thresholding and if a second study on the same stimulation paradigm would utilize another method of analysis divergent results would occur. Considering that in this field methods of analysis and statistics have proliferated, it is paramount to avoid a scattered picture of the results gained by fMRI (Kriegeskorte et al., 2009). The Bi-CoPaM paradigm allows us to integrate many analysis methods and to obtain robust and reproducible clusters from various datasets. The Bi-CoPaM has been successfully applied in biology to various gene clustering applications, in which genes represent the objects to be clustered and gene expression represent their quantified features. For instance, its application to two yeast cell-cycle datasets revealed new insights regarding a poorly understood gene in yeast (Abu-Jamous et al., 2013b), and its application to forty diverse yeast datasets identified a novel cluster of genes consistently anti-correlated with growth (Abu-Jamous et al., 2014b). In our previous study (Liu et al., 2017), we pointed out that different clustering algorithms (K-mean, SOM and hierarchical) produce partly divergent sets of clustering results, whereas Bi-CoPaM generates consensus among them, thus reducing the risks of capturing artifacts. Furthermore, the previous study (Liu et al., 2017) and current results show non-trivial clusters covering large continuous brain regions, confirming the robustness of the method. Remarkably, unlike some algorithms that artificially introduce the spatial constraints to the clustering generation process (Craddock et al., 2012; Blumensath et al., 2013), our spatial information-free strategy guaranteed that the voxels in fMRI data were clustered purely based on the similarities of their BOLD time series rather than on their topologies in the brain. It should be mentioned though that, similarly to other methods, our current approach for studying functional connectivity does not provide information on the temporal succession of increased connectivity in each obtained cluster.

The first finding of this study is the separation of clusters of correlated neural activity between the three experimental conditions. The conditions not requiring an explicit evaluation of liking of the music excerpts were most similar to each other in terms of shared voxels in the resulting clusters as evidenced by the topology interaction analyses. More specifically, the naturalistic listening condition and the liking judgment condition showed similar functional connectivity only between visual areas (particularly parts of the bilateral middle and superior occipital gyri). Also, the liking judgment condition showed similarly correlated neural activity to the gender judgment condition only in fronto-parietal areas related to action observation (particularly the right postcentral gyrus and the pars opercularis of the left inferior frontal gyrus). In contrast, the naturalistic listening condition shared four clusters with the gender judgment condition, meaning that they had similar increased connectivity between auditory (temporal pole, bilateral superior and middle temporal gyri, Heschl's gyrus), frontal (orbital part of inferior frontal gyrus) cerebral areas, cerebellum, and limbic areas (parahippocampal gyrus, amygdala, insula, thalamus, hippocampus).

When comparing the clusters obtained for each of the three experimental conditions, the connectivity of motor-related areas with the Rolandic operculum was much more evident for the gender judgment condition as opposed to the liking judgment condition, with only one shared voxel between the two. While the Rolandic operculum has been related to musical pleasure in previous studies (Koelsch et al., 2006; Green et al., 2012), it is also implicated in both overt and covert singing and speaking (Wildgruber et al., 1996; Riecker et al., 2000; Jeffries et al., 2003). One can thus speculate that the focus on the vocal properties of the song excerpts used here as stimuli would have prompted participants to recruit sound production planning areas of the brain.

Chatterjee and Vartanian (2016) recently proposed that all art phenomena emerge from the interaction between three main mental/neural systems: a sensory-motor one (sensation, perception, motor system), a knowledge-meaning one (expertise, context, culture) and an emotion-evaluation one (reward, emotion, wanting/liking). Also Juslin (2013) viewed aesthetic judgment as the final outcome of a summation of different emotion-inductive mechanisms. In previous works (Nieminen et al., 2011; Brattico and Pearce, 2013; Brattico et al., 2013; Reybrouck and Brattico, 2015), authors proposed a detailed spatiotemporal road map of music aesthetic processes in the brain. They suggested a distinction between unconscious, low-level perceptual-emotional stages and reflective processes involving cognitive control and leading to the three main outcomes of an aesthetic experience, namely emotion, preference and judgment. The early and late emotional processes during a musical experience can be modulated by what Hodges (2016) has termed “focus,” namely the act of paying attention to the music aimed at reaching an aesthetic evaluation of it. Here and in previous work (Brattico et al., 2013), we extended this concept to that of an internal state predisposing to attentive watching/listening in the case of performance arts or contemplation in the case of static arts, inspired by previous proposals (Hargreaves et al., 2012; Bundgaard, 2015). Based on these premises, we here hypothesized that the individual's psychological state or internal context is an important predictor of the emotion-related brain processes occurring during music listening. Our findings for the liking judgment condition of connected regions within the ventral and dorsal attention networks, including parietal regions (bilateral supramarginal and angular gyri), frontal regions, including the ones related to the action observation system (bilateral precentral and postcentral gyri, supplementary motor area, cingulate cortex), and visual structures (bilateral middle, superior occipital gyri, cuneus, lingual gyrus) are in line with the role of focused attention and action observation during aesthetic music listening (Molnar-Szakacs and Overy, 2006; D'Ausilio, 2009). Moreover, we speculate that, after an initial fast reaction to positively or negatively valenced sounds, attentional processes render the sounds available for conscious appraisal and evaluation, as in the liking judgment condition. Relevant to this, a previous study (Bogert et al., 2016) contrasted two conditions involving recognition of sad, happy, or fearful emotions in the music stimuli and descriptive judgments of the same musical material. The authors of the study reported maximal fMRI signal in fronto-parietal and occipital brain structures (such as bilateral superior frontal gyrus, inferior occipital gyrus, lingual gyrus, fusiform gyrus) specifically for the condition requiring the explicit classification of the emotions perceived in the music. While Bogert et al. (2016) only analyzed regional activations with univariate general linear model, the pattern of findings lets us suppose that the functional network of co-varying activity would be comparable to the one observed in the current study. The evidence is divergent for visual art stimulation: neural responses related to affective processes were obtained only when participants focused on giving an evaluative beauty judgment of abstract black-white stimuli and not when they were passively contemplating the stimuli (Höfel and Jacobsen, 2007a). Hence, based on these studies, it seems that deciding to like or dislike a tune has the effect of down-regulating subcortical emotion-related neural activity, while the effect is opposite for visual art.

When considering the connectivity between several occipital regions observed here during the explicit liking condition, and, to a lesser extent, to the naturalistic listening condition, one cannot immediately reject the alternative explanation that our data-driven clustering analysis evidenced those regions that are processing the visual information during the experimental tasks, since participants had eyes open. However, the absence of occipital clusters from the gender judgment condition rather suggests a functional role of those clusters that goes beyond basic visual processing, and that might instead relate to focused attentional processes during emotional music listening (Bogert et al., 2016).

In turn, the condition in which participants were asked to explicitly decide on the descriptive, non-evaluative aspects of the music excerpts, namely whether they contained one, few or many instruments, elicited more subcortical neural structures such as in the caudate, pallidum, and cortical areas previously linked to emotion processing, such as the inferior parietal lobule (see also Flores-Gutiérrez et al., 2007; Chapin et al., 2010; Satoh et al., 2011). Also in our study, the connected regions activated by the gender judgment task have been formerly clearly related to emotion processing: parahippocampal gyrus, amygdala, insula, hippocampus, thalamus, medial orbitofrontal cortex, caudate nucleus, and the vermis of the cerebellum. This limbic network is here closely communicating with ventral stream auditory regions such as the anterior superior temporal gyrus. A second network involved with the gender judgment task included sensorimotor regions coupled with the bilateral insula and the right putamen. Naturalistic listening produced coupled activity in several overlapping regions compared to those elicited by the gender judgment condition. However, naturalistic listening was associated with a more scattered connectivity pattern as compared with the gender judgment condition, displaying six separate clusters over attention-, perception- and emotion-related areas. Overall, several studies showed that the degree of connectivity between striatal areas, ventrolateral prefrontal regions and auditory cortices is crucial for determining the subjective degree of enjoyment of a musical piece (Blood and Zatorre, 2001; Salimpoor et al., 2013; Martínez-Molina et al., 2016; Sachs et al., 2016).

Notably, the activity and connectivity of sensorimotor areas (such as the precentral and postcentral gyri and the supplementary motor area) found for all the three experimental conditions and particularly for the gender judgment condition, have been consistently observed in response to music-induced emotions (Blood and Zatorre, 2001; Mitterschiffthaler et al., 2007; Brattico et al., 2015; Bogert et al., 2016). Furthermore, the connected areas found here (particularly the opercular part of the inferior frontal gyrus and the inferior parietal lobule) partially overlap with the action observation network (or “mirror neuron” system), activated both by motor production by an individual and by the perception of motor acts by others (Rizzolatti et al., 1996; Morin and Grèzes, 2008). Some proposed theories in music psychology argue that motor mimicking of sounds resembling an emotional vocalization is a crucial mechanism for inducing emotions (Juslin and Västfjäll, 2008; Juslin, 2013).

In conclusion, this study is an initial attempt to apply a novel clustering paradigm to aesthetic research. Developments of this approach should involve applications of the Bi-CoPaM paradigm to other clustering methods than the ones used here. In particular, independent component analysis (ICA) could be considered since it is increasingly used in network neuroscience, including music research (Cong et al., 2014; Burunat et al., 2017). Moreover, other partitioning procedures, such as Q cut by Ruan and Zhang (2008), modularity approaches by Newman and Girvan (2004), along with partitioning of scaled inclusivity for finding voxel-based partitioning consistency across groups (Steen et al., 2011), could be applied in future to provide useful information on the functional connectivity during music-related processes.

Moreover, the current findings demonstrated clearly that explicit judgments of the hedonic value of a musical piece are important in shaping neural connectivity to music, and specifically in connecting brain regions related to attention and cognition. In turn, when the participant is focusing on judging non-evaluative aspects of the music, areas related to emotions and pleasures become more coupled. Here it is relevant to state that the findings were obtained with music unfamiliar to the participants. Based on previous findings obtained with another paradigm (Brattico et al., 2015), we might expect divergent effects with highly familiar music. Hence, the study was successful in isolating the responses of the limbic and pleasure neural circuits during spontaneous music listening, from the responses of the cognitive and attentional frontoparietal circuits during conscious liking of music. The findings are in line with previous models of aesthetic pleasure, where core pleasure, or the fast, reflective reactions to stimuli involving subcortical brain processes, is distinct from conscious liking. For the latter, higher-order cortical structures are required to appraise the outcome of the subcortical pleasure centers, in order to finally issue an explicit evaluative judgment (Brattico, 2015; Chatterjee and Vartanian, 2016; Kringelbach and Berridge, 2017).

## Author contributions

CL: Data processing and analysis; write manuscript. EB: fMRI experiment design; write manuscript; study director. BA-j: Data analysis; write manuscript. CP: fMRI experiment design; data collection. TJ: fMRI experiment design; study director. AN: study director, coordinator, write manuscript.

### Conflict of interest statement

The authors declare that the research was conducted in the absence of any commercial or financial relationships that could be construed as a potential conflict of interest.
