# Supplementary material for: Effect of Explicit Evaluation on Neural Connectivity Related to Listening to Unfamiliar Music
Source: Front Hum Neurosci. 2017 Dec 19;11:611. doi: 10.3389/fnhum.2017.00611 (PMC5742221; doi:10.3389/fnhum.2017.00611)
Supplement: Supplementary file 1 [file DataSheet1.DOCX]

Supplementary Material

1. Bi-CoPaM paradigm.

The Bi-CoPaM method is a tunable consensus clustering paradigm which can utilize various single clustering algorithms as well as a number of different datasets to identify the subset of objects (e.g. voxels) that are consistently correlated. When one clustering method is applied to one dataset, it produces one partition (which is a set of clusters). Given C clustering methods and L datasets, the Bi-CoPaM executes the following four main steps:

1. Partition generation: each of the C clustering methods is applied to each of the L datasets yielding R (= C × L) partitions.
2. Relabelling: because clustering is unsupervised, there are no labels for the clusters in the different partitions, i.e. the i-th cluster in one partition is not guaranteed to match the i-th cluster in another partition. Relabelling reorders the clusters in the partitions so that they become aligned. Min–min approach was used to perform relabelling.
3. Fuzzy consensus partition matrix (CoPaM) generation: the relabelled partitions are averaged to produce a fuzzy CoPaM in which each voxel has a fuzzy membership value in each of the clusters based on the number of individual partitions that assigned it to it.
4. Binarization/Quenching: the fuzzy CoPaM is binarized to produce the final binary partition. Different threshold binarization (DTB) technique assigns a voxel to a cluster if and only if its fuzzy membership value in that cluster is higher than its closest competing cluster fuzzy membership value by the value of the parameter δ. The parameter δ ∈ [0,1] controls the tightness of the cluster where δ = 0.0 is the least tight (most sparse) and δ = 1.0 is the tightest.

The readers are advised to read the original method paper on Bi-CoPaM and its adaptation for fMRI data:

Paradigm of tunable clustering using binarization of consensus partition matrices (Bi-CoPaM) for gene discovery. B Abu-Jamous, R Fa, DJ Roberts, AK Nandi. PLoS One, 2013.

Chao Liu, Basel Abu-Jamous, Elvira Brattico, and Asoke K. Nandi. Towards Tunable Consensus Clustering for Studying Functional Brain Connectivity During Affective Processing. International Journal of Neural Systems 2017.

1. Min-Min relabelling approach

Relabelling a partition matrix $\boldsymbol{U}$ to be aligned with a reference partition matrix $\boldsymbol{U}^{ref}$ aims at finding a matrix $\hat{\boldsymbol{U}}$ which represents one of the permutations of the rows of $\boldsymbol{U}$ such that its similarity to $\boldsymbol{U}^{ref}$ is maximised. Finding the optimal labelling correspondence is an NP-complete combinatorial problem, which has a search space of $K!$, making exhaustive search impractical for any large and even not so large value of $K$. Thus, the heuristic algorithms, e.g. min-min method, are often used to approximate the solution. The min-min relabelling method consists of four steps:

**Step 1**. A dissimilarity matrix $\boldsymbol{S}_{K\times K}$ is constructed with pairwise Euclidian distances between the rows (clusters) of the matrix $\boldsymbol{U}$ and the rows of the reference matrix $\boldsymbol{U}^{ref}$.

**Step 2**. The minimum value in each column of $\boldsymbol{S}$ is found, followed by identifying the minimum value of these minima. Then the rows (clusters) from $\boldsymbol{U}$ and $\boldsymbol{U}^{ref}$ which correspond to this dissimilarity value are mapped to each other, i.e. these two clusters are considered to have the same cluster label.

**Step 3**. The row and the column that intersect at the aforementioned dissimilarity value are deleted from the similarity matrix $\boldsymbol{S}$. Then the minimum of the column’s minima in the reduced matrix is further identified leading to mapping next pair of clusters from $\boldsymbol{U}$ and $\boldsymbol{U}^{ref}$.

**Step 4**. Repeat step 2 and 3 until all rows (clusters) from $\boldsymbol{U}$ are mapped to their corresponding rows (clusters) in $\boldsymbol{U}^{ref}$.

1. MN scatter plot technique.

The Bi-CoPaM method requires pre-setting the number of clusters (K). Moreover, the tightening parameter δ needs to be optimized. M-N scatter plots technique tackles those issues. Each time that Bi-CoPaM is applied with different K values, DTB binarisation is performed with a range of δ values (e.g., from 0.0 to 1.0 with 0.1 steps). All of the individual clusters that appear in the results are scattered on a 2-D plot where the horizontal axis (M) represents the average mean square error (MSE) values of the cluster over all of the datasets, and the vertical axis (N) represents the logarithm of the number of voxels in the cluster. Both axes are normalized to have unity length. The average MSE per voxel for the k-th cluster can be calculated by

$$MSE_{cluster(k)}=\frac{1}{LN_{k}}\sum_{l=1}^{L} \sum_{n\in C_{k}} \left\| \boldsymbol{x}_{n}^{l}-\boldsymbol{z}_{k}^{l} \right\|^{2}$$

where L is the number of datasets, $N_{k}$ is the number of voxels in the k-th cluster, $C_{k}$ is the set of voxels in the k-th cluster, $x_{n}^{l}$ is the normalized BOLD signal vector of the n-th voxel in the cluster from the l-th dataset, $z_{k}^{l}$ is the average normalized BOLD signal vector of the voxels in the k-th cluster from the l-th dataset, and $\left\| . \right\|$ is the norm of a vector. The objective is to maximize the number of voxels included in the clusters while minimizing the dispersion within the clusters measured by the MSE metric.

As all of the individual clusters generated under all of the different combinations of parameters are scattered on this M-N plot as shown in Figure 1.

Figure 1. Example of M-N scatter plots technique.

The cluster closest to the top left corner of the plot is selected as the top cluster (blue dot). This cluster is expected to be large with many voxels (high vertical axis value), yet tight with high correlation (low MSE value on the horizontal axis). The selected cluster and all of the other clusters that have overlaps with it, even by a single voxel, are removed from the plot. Then, the closest remaining cluster to the top left corner of the plot is selected as the second best distinct cluster. The steps of selecting clusters and removing those with overlaps with the selected ones are repeated iteratively up to a pre-set maximum number of clusters or earlier when the scatter plots are empty. The final number of clusters is not predetermined as it depends on when the plot becomes completely empty. Moreover, the produced clusters are ordered in a descending manner regarding their tightness and size measured by their closeness to the top left corner. Practically, the higher ranked clusters are of interest to the downstream analysis while most of the low ranked clusters may be considered as containers of irrelevant voxels and are thus discarded.

1. List of stimuli used in the listening experiment
