# Supplementary material for: Effect of Explicit Evaluation on Neural Connectivity Related to Listening to Unfamiliar Music
Source: Front Hum Neurosci. 2017 Dec 19;11:611. doi: 10.3389/fnhum.2017.00611 (PMC5742221; doi:10.3389/fnhum.2017.00611)
Supplement: Supplementary file 2 [file DataSheet2.docx]

**Appendix**

**Tables of cluster information. Please note that those 1-2 voxels are not a cluster, but included in a certain cluster. For example, a cluster might consist multiple anatomical regions, some of them includes hundred voxels but some are very small with only several voxels. Set operation symbols are used to denote the relationship between any two clusters. For example, C5_L - C5_N ∩C5_L means: the voxels only belonging to Cluster 5 in liking judgement session, when Cluster 5 in liking session has an intersection with Cluster 5 in Naturalistic listening. In table 4-6, the letter i is short for intersection, as in the Figure 5-7, the circle is dense and putting the complete relationship denotation will make the figure unreadable. A bracket with the complete denotation for an intersection area will appear in table 4-6 but not in figure 5-7.**

Table 1. Anatomical information for clusters in liking judgment session.

|  |  |  |  |  |
| --- | --- | --- | --- | --- |
|  | C3(red) |  |  |  |
|  | Anatomical labels | MNI coordinates | Size |  |
|  |  | [x, y, z] | [voxels] |  |
|  | 'Supramarginal gyrus (R)' | [59,-28,32] | 195 |  |
|  | 'Postcentral gyrus (R)' | [46,-26,44] | 162 |  |
|  | 'Postcentral gyrus (L)' | [-53,-21,37] | 133 |  |
|  | 'Supramarginal gyrus (L)' | [-58,-28,33] | 64 |  |
|  | 'Supplementary motor area (R)' | [6,5,52] | 75 |  |
|  | 'Inferior parietal, but supramarginal and angular gyri (L)' | [-49,-27,42] | 53 |  |
|  | 'Inferior frontal gyrus, opercular part (L) ' | [-55,9,16] | 35 |  |
|  | 'Middle temporal gyrus (L)' | [-53,-55,6] | 67 |  |
|  | 'Precentral gyrus (R)' | [48,-3,40] | 45 |  |
|  | 'Rolandic operculum (R)' | [51,2,13] | 28 |  |
|  | 'Inferior parietal, but supramarginal and angular gyri (R)' | [45,-38,49] | 25 |  |
|  | 'Supplementary motor area (L)' | [-1,1,52] | 24 |  |
|  | 'Median cingulate and paracingulate gyrus (R)' | [4,8,42] | 10 |  |
|  | 'Precentral gyrus (L)' | [-55,6,26] | 8 |  |
|  | 'Rolandic operculum (L)' | [-54,4,12] | 1 |  |
|  | 'Median cingulate and paracingulate gyrus (L)' | [-1,0,47] | 2 |  |
|  |  |  |  |  |
|  | C5 (blue) |  |  |  |
|  | Anatomical labels | MNI coordinates | Size |  |
|  |  | [x, y, z] | [voxels] |  |
|  | 'Middle occipital gyrus (R)' | [33,-86,9] | 143 |  |
|  | 'Middle occipital gyrus (L)' | [-38,-82,12] | 162 |  |
|  | 'Superior occipital gyrus (R)' | [23,-89,20] | 68 |  |
|  | 'Fusiform gyrus (L)' | [-29,-68,-12] | 68 |  |
|  | 'Inferior occipital gyrus (R)' | [41,-81,-6] | 44 |  |
|  | 'Superior occipital gyrus (L)' | [-18,-91,32] | 25 |  |
|  | 'Cuneus (R)' | [18,-96,11] | 15 |  |
|  | 'Calcarine fissure and surrounding cortex (R)' | [7,-88,4] | 17 |  |
|  | 'Fusiform gyrus (R)' | [30,-72,-11] | 20 |  |
|  | 'Inferior occipital gyrus (L)' | [-33,-76,-9] | 10 |  |
|  | 'Lingual gyrus (L)' | [-22,-72,-12] | 13 |  |
|  | 'Lingual gyrus (R)' | [25,-66,-1] | 10 |  |
|  | 'Calcarine fissure and surrounding cortex (L)' | [4,-90,5] | 5 |  |
|  | 'Cuneus (L)' | [-10,-89,37] | 2 |  |
|  |  |  |  |  |

Table 2. Anatomical information for clusters in gender judgment session.

|  |  |  |  |  |
| --- | --- | --- | --- | --- |
|  | C3(red) |  |  |  |
|  | Anatomical labels | MNI coordinates | Size |  |
|  |  | [x, y, z] | [voxels] |  |
|  | 'Temporal pole, superior temporal gyrus (L)' | [-40,12,-22] | 596 |  |
|  | 'Temporal pole, superior temporal gyrus (R)' | [41,12,-23] | 409 |  |
|  | 'Crus I of cerebellum (L)' | [-30,-80,-25] | 374 |  |
|  | 'Parahippocampal gyrus (L)' | [-17,-10,-22] | 234 |  |
|  | 'Superior temporal gyrus (L)' | [-48,-5,-8] | 256 |  |
|  | 'Lobule III of cerebellum (R)' | [12,-34,-19] | 88 |  |
|  | 'Vermis III of cerebellum' | [2,-40,-12] | 77 |  |
|  | 'Crus I of cerebellum (R)' | [40,-76,-28] | 218 |  |
|  | 'Temporal pole, middle temporal gyrus (L)' | [-42,14,-31] | 126 |  |
|  | 'Lobule III of cerebellum (L)' | [-9,-37,-18] | 67 |  |
|  | 'Lobules IV-V of cerebellum (L)' | [-12,-39,-14] | 149 |  |
|  | 'Parahippocampal gyrus (R)' | [21,-3,-24] | 134 |  |
|  | 'Amygdala (L)' | [-22,0,-20] | 61 |  |
|  | 'Insula (R)' | [40,3,-8] | 139 |  |
|  | 'Temporal pole, middle temporal gyrus (R)' | [44,14,-29] | 83 |  |
|  | 'Vermis IV-V of cerebellum' | [1,-55,-1] | 64 |  |
|  | 'Amygdala (R)' | [29,1,-24] | 34 |  |
|  | 'Hippocampus (L)' | [-19,-17,-16] | 62 |  |
|  | 'Thalamus (L)' | [-3,-21,8] | 66 |  |
|  | 'Vermis I-II of cerebellum' | [1,-38,-20] | 8 |  |
|  | 'Inferior frontal gyrus, orbital part (R)' | [36,23,-18] | 55 |  |
|  | 'Crus II of cerebellum (L)' | [-8,-87,-26] | 59 |  |
|  | 'Olfactory cortex (L)' | [0,12,-9] | 15 |  |
|  | 'Vermis VI of cerebellum' | [-1,-73,-10] | 17 |  |
|  | 'Lobules IV-V of cerebellum (R)' | [10,-44,-9] | 27 |  |
|  | 'Gyrus rectus (L)' | [0,28,-18] | 21 |  |
|  | 'Olfactory cortex (R)' | [30,10,-20] | 1 |  |
|  | 'Gyrus rectus (R)' | [1,23,-17] | 4 |  |
|  | 'Inferior temporal gyrus (L)' | [-56,-55,-24] | 31 |  |
|  | 'Hippocampus (R)' | [21,-3,-21] | 3 |  |
|  | 'Caudate nucleus (L)' | [-2,15,0] | 2 |  |
|  | 'Fusiform gyrus (L)' | [-18,-35,-19] | 8 |  |
|  | 'Inferior temporal gyrus (R)' | [54,-55,-26] | 13 |  |
|  |  |  |  |  |
|  | C5(blue) |  |  |  |
|  | Anatomical labels | MNI coordinates | Size |  |
|  |  | [x, y, z] | [voxels] |  |
|  | 'Insula (R)' | [41,-2,3] | 415 |  |
|  | 'Superior temporal gyrus (R)' | [55,-24,6] | 662 |  |
|  | 'Superior temporal gyrus (L)' | [-53,-18,4] | 405 |  |
|  | 'Rolandic operculum (R)' | [49,-10,13] | 231 |  |
|  | 'Rolandic operculum (L)' | [-47,-11,10] | 182 |  |
|  | 'Heschl''s gyrus (R)' | [45,-18,9] | 108 |  |
|  | 'Heschl''s gyrus (L)' | [-43,-18,9] | 72 |  |
|  | 'Insula (L)' | [-42,0,0] | 158 |  |
|  | 'Middle temporal gyrus (L)' | [-56,-25,-1] | 233 |  |
|  | 'Postcentral gyrus (R)' | [57,-15,38] | 181 |  |
|  | 'Inferior frontal gyrus, opercular part (R)' | [49,14,11] | 88 |  |
|  | 'Inferior frontal gyrus, opercular part (L) ' | [-55,10,11] | 72 |  |
|  | 'Precentral gyrus (R)' | [58,2,33] | 115 |  |
|  | 'Supramarginal gyrus (R)' | [53,-31,25] | 56 |  |
|  | 'Supplementary motor area (R)' | [9,-3,63] | 30 |  |
|  | 'Putamen (R)' | [33,7,7] | 3 |  |
|  |  |  |  |  |

Table 3. Anatomical information for clusters in naturalistic listening.

|  |  |  |  |  |
| --- | --- | --- | --- | --- |
|  | C4(red) |  |  |  |
|  | Anatomical labels | MNI coordinates | Size |  |
|  |  | [x, y, z] | [voxels] |  |
|  | 'Middle frontal gyrus, orbital part (R)' | [33,52,-14] | 88 |  |
|  | 'Inferior frontal gyrus, orbital part (R)' | [39,31,-18] | 77 |  |
|  | 'Inferior frontal gyrus, orbital part (L)' | [-40,37,-16] | 76 |  |
|  | 'Middle frontal gyrus, orbital part (L)' | [-27,56,-13] | 49 |  |
|  | 'Gyrus rectus (R)' | [5,56,-20] | 31 |  |
|  | 'Temporal pole, middle temporal gyrus (L)' | [-48,15,-30] | 27 |  |
|  | 'Superior frontal gyrus, orbital part (R)' | [13,57,-19] | 23 |  |
|  | 'Superior frontal gyrus, orbital part (L)' | [-15,60,-15] | 23 |  |
|  | 'Gyrus rectus (L)' | [-3,59,-21] | 19 |  |
|  | 'Inferior frontal gyrus, medial orbital (R)' | [7,66,-12] | 17 |  |
|  | 'Inferior frontal gyrus, medial orbital (L)' | [-3,65,-10] | 8 |  |
|  | 'Middle temporal gyrus (L)' | [-60,-1,-27] | 20 |  |
|  | 'Inferior temporal gyrus (L)' | [-57,-4,-29] | 7 |  |
|  |  |  |  |  |
|  | C5(blue) |  |  |  |
|  | Anatomical labels | MNI coordinates | Size |  |
|  |  | [x, y, z] | [voxels] |  |
|  | 'Calcarine fissure and surrounding cortex (L)' | [-7,-74,10] | 227 |  |
|  | 'Calcarine fissure and surrounding cortex (R)' | [11,-76,8] | 166 |  |
|  | 'Cuneus (R)' | [11,-83,23] | 135 |  |
|  | 'Cuneus (L)' | [-3,-82,21] | 101 |  |
|  | 'Lingual gyrus (R)' | [13,-65,1] | 72 |  |
|  | 'Superior occipital gyrus (R)' | [20,-89,22] | 54 |  |
|  | 'Middle occipital gyrus (L)' | [-34,-83,18] | 78 |  |
|  | 'Lingual gyrus (L)' | [-10,-67,0] | 63 |  |
|  | 'Superior occipital gyrus (L)' | [-19,-89,26] | 49 |  |
|  | 'Fusiform gyrus (L)' | [-29,-70,-11] | 25 |  |
|  | 'Middle occipital gyrus (R)' | [29,-81,16] | 4 |  |
|  | 'Inferior occipital gyrus (L)' | [-32,-78,-12] | 1 |  |
|  |  |  |  |  |
|  | C6(green) |  |  |  |
|  | Anatomical labels | MNI coordinates | Size |  |
|  |  | [x, y, z] | [voxels] |  |
|  | 'Temporal pole, superior temporal gyrus (L)' | [-37,10,-23] | 153 |  |
|  | 'Parahippocampal gyrus (L)' | [-16,-10,-23] | 107 |  |
|  | 'Lobule III of cerebellum (R)' | [12,-33,-19] | 53 |  |
|  | 'Temporal pole, superior temporal gyrus (R)' | [39,9,-22] | 83 |  |
|  | 'Lobule III of cerebellum (L)' | [-11,-35,-20] | 31 |  |
|  | 'Superior temporal gyrus (L)' | [-46,-6,-9] | 63 |  |
|  | 'Lobules IV-V of cerebellum (L)' | [-12,-36,-17] | 44 |  |
|  | 'Vermis III of cerebellum' | [1,-39,-12] | 24 |  |
|  | 'Parahippocampal gyrus (R)' | [19,-7,-21] | 41 |  |
|  | 'Amygdala (L)' | [-21,1,-21] | 15 |  |
|  | 'Insula (R)' | [42,6,-10] | 33 |  |
|  | 'Vermis I-II of cerebellum' | [0,-37,-19] | 5 |  |
|  | 'Amygdala (R)' | [29,3,-27] | 6 |  |
|  | 'Hippocampus (L)' | [-14,-6,-21] | 9 |  |
|  | 'Temporal pole, middle temporal gyrus (L)' | [-40,9,-28] | 5 |  |
|  | 'Olfactory cortex (L)' | [-2,10,-14] | 1 |  |
|  | 'Lingual gyrus (L)' | [-11,-36,-7] | 7 |  |
|  |  |  |  |  |
|  | C8(violet) |  |  |  |
|  | Anatomical labels | MNI coordinates | Size |  |
|  |  | [x, y, z] | [voxels] |  |
|  | 'Insula (R)' | [41,20,-3] | 29 |  |
|  | 'Anterior cingulate and paracingulate gyrus (L)' | [-1,37,8] | 27 |  |
|  | 'Inferior frontal gyrus, orbital part (R)' | [41,29,-7] | 23 |  |
|  | 'Inferior frontal gyrus, triangular part (R)' | [47,24,4] | 24 |  |
|  | 'Inferior frontal gyrus, triangular part (L)' | [-46,22,2] | 24 |  |
|  | 'Inferior frontal gyrus, opercular part (R)' | [51,15,5] | 19 |  |
|  | 'Anterior cingulate and paracingulate gyrus (R)' | [3,41,7] | 7 |  |
|  | 'Inferior frontal gyrus, orbital part (L)' | [-45,22,-4] | 3 |  |
|  | 'Insula (L)' | [-39,22,1] | 2 |  |
|  |  |  |  |  |
|  | C9(yellow) |  |  |  |
|  | Anatomical labels | MNI coordinates | Size |  |
|  |  | [x, y, z] | [voxels] |  |
|  | 'Superior temporal gyrus (R)' | [57,-17,3] | 177 |  |
|  | 'Superior temporal gyrus (L)' | [-55,-11,2] | 69 |  |
|  | 'Middle temporal gyrus (L)' | [-59,-27,-2] | 39 |  |
|  | 'Insula (R)' | [43,-9,-1] | 24 |  |
|  | 'Heschl''s gyrus (R)' | [46,-19,7] | 11 |  |
|  | 'Heschl''s gyrus (L)' | [-50,-16,7] | 2 |  |
|  |  |  |  |  |
|  | C11(cyan) |  |  |  |
|  | Anatomical labels | MNI coordinates | Size |  |
|  |  | [x, y, z] | [voxels] |  |
|  | 'Angular gyrus (R)' | 121 | [50,-59,32] |  |
|  | 'Middle frontal gyrus (R)' | 20 | [31,16,46] |  |
|  | 'Inferior parietal, but supramarginal and angular gyri (R)' | 11 | [52,-58,44] |  |
|  |  |  |  |  |

Table 4. Cluster topology interaction between liking judgment and gender judgment.

|  |  |  |  |  |
| --- | --- | --- | --- | --- |
|  | C5_G ∩ C3_L |  |  |  |
|  | Anatomical labels | MNI coordinates | Size |  |
|  |  | [x, y, z] | [voxels] |  |
|  | 'Postcentral gyrus (R)' | [54,-20,35] | 13 |  |
|  | 'Inferior frontal gyrus, opercular part (L) ' | [-57,8,12] | 7 |  |
|  | 'Supramarginal gyrus (R)' | [55,-26,29] | 3 |  |
|  | 'Precentral gyrus (R)' | [58,4,26] | 2 |  |
|  | 'Rolandic operculum (R)' | [54,4,16] | 1 |  |
|  | 'Supplementary motor area (R)' | [10,-2,58] | 1 |  |
|  |  |  |  |  |
|  | C5_G − C5_G ∩ C3_L |  |  |  |
|  | Anatomical labels | MNI coordinates | Size |  |
|  |  | [x, y, z] | [voxels] |  |
|  | 'Insula (R)' | [41,-2,3] | 415 |  |
|  | 'Superior temporal gyrus (R)' | [55,-24,6] | 662 |  |
|  | 'Superior temporal gyrus (L)' | [-53,-18,4] | 405 |  |
|  | 'Rolandic operculum (R)' | [49,-10,13] | 230 |  |
|  | 'Rolandic operculum (L)' | [-47,-11,10] | 182 |  |
|  | 'Heschl''s gyrus (R)' | [45,-18,9] | 108 |  |
|  | 'Heschl''s gyrus (L)' | [-43,-18,9] | 72 |  |
|  | 'Insula (L)' | [-42,0,0] | 158 |  |
|  | 'Middle temporal gyrus (L)' | [-56,-25,-1] | 233 |  |
|  | 'Postcentral gyrus (R)' | [57,-14,39] | 168 |  |
|  | 'Inferior frontal gyrus, opercular part (R)' | [49,14,11] | 88 |  |
|  | 'Inferior frontal gyrus, opercular part (L) ' | [-54,10,11] | 65 |  |
|  | 'Precentral gyrus (R)' | [58,2,33] | 113 |  |
|  | 'Supramarginal gyrus (R)' | [53,-31,24] | 53 |  |
|  | 'Supplementary motor area (R)' | [9,-4,63] | 29 |  |
|  | 'Putamen (R)' | [33,7,7] | 3 |  |
|  |  |  |  |  |
|  | C5_G − C5_G ∩ C3_L |  |  |  |
|  | Anatomical labels | MNI coordinates | Size |  |
|  |  | [x, y, z] | [voxels] |  |
|  | 'Supramarginal gyrus (R)' | [59,-28,32] | 192 |  |
|  | 'Postcentral gyrus (R)' | [45,-26,44] | 149 |  |
|  | 'Postcentral gyrus (L)' | [-53,-21,37] | 133 |  |
|  | 'Supramarginal gyrus (L)' | [-58,-28,33] | 64 |  |
|  | 'Supplementary motor area (R)' | [6,5,52] | 74 |  |
|  | 'Inferior parietal, but supramarginal and angular gyri (L)' | [-49,-27,42] | 53 |  |
|  | 'Middle temporal gyrus (L)' | [-53,-55,6] | 67 |  |
|  | 'Inferior frontal gyrus, opercular part (L) ' | [-54,9,17] | 28 |  |
|  | 'Precentral gyrus (R)' | [48,-4,41] | 43 |  |
|  | 'Rolandic operculum (R)' | [50,2,13] | 27 |  |
|  | 'Inferior parietal, but supramarginal and angular gyri (R)' | [45,-38,49] | 25 |  |
|  | 'Supplementary motor area (L)' | [-1,1,52] | 24 |  |
|  | 'Median cingulate and paracingulate gyrus (R)' | [4,8,42] | 10 |  |
|  | 'Precentral gyrus (L)' | [-55,6,26] | 8 |  |
|  | 'Rolandic operculum (L)' | [-54,4,12] | 1 |  |
|  | 'Median cingulate and paracingulate gyrus (L)' | [-1,0,47] | 2 |  |
|  |  |  |  |  |

Table 5. Cluster topology interaction between liking judgment and naturalistic listening.

|  |  |  |  |  |
| --- | --- | --- | --- | --- |
|  | C5_N ∩ C5_L |  |  |  |
|  | Anatomical labels | MNI coordinates | Size |  |
|  |  | [x, y, z] | [voxels] |  |
|  | 'Superior occipital gyrus (R)' | [21,-91,21] | 13 |  |
|  | 'Middle occipital gyrus (L)' | [-38,-80,16] | 9 |  |
|  | 'Fusiform gyrus (L)' | [-29,-74,-11] | 4 |  |
|  | 'Calcarine fissure and surrounding cortex (R)' | [7,-90,4] | 3 |  |
|  | 'Middle occipital gyrus (R)' | [29,-80,16] | 2 |  |
|  | 'Superior occipital gyrus (L)' | [-22,-90,30] | 1 |  |
|  | 'Lingual gyrus (R)' | [22,-70,2] | 1 |  |
|  |  |  |  |  |
|  | C5_N − C5_N ∩ C5_L |  |  |  |
|  | Anatomical labels | MNI coordinates | Size |  |
|  |  | [x, y, z] | [voxels] |  |
|  | 'Calcarine fissure and surrounding cortex (L)' | [-7,-74,10] | 227 |  |
|  | 'Calcarine fissure and surrounding cortex (R)' | [11,-76,8] | 163 |  |
|  | 'Cuneus (R)' | [11,-83,23] | 135 |  |
|  | 'Cuneus (L)' | [-3,-82,21] | 101 |  |
|  | 'Lingual gyrus (R)' | [13,-65,1] | 71 |  |
|  | 'Lingual gyrus (L)' | [-10,-67,0] | 63 |  |
|  | 'Superior occipital gyrus (L)' | [-19,-89,26] | 48 |  |
|  | 'Middle occipital gyrus (L)' | [-34,-83,18] | 69 |  |
|  | 'Superior occipital gyrus (R)' | [20,-88,23] | 41 |  |
|  | 'Fusiform gyrus (L)' | [-29,-70,-11] | 21 |  |
|  | 'Inferior occipital gyrus (L)' | [-32,-78,-12] | 1 |  |
|  | 'Middle occipital gyrus (R)' | [29,-81,15] | 2 |  |
|  |  |  |  |  |
|  | C5_L − C5_N ∩ C5_L |  |  |  |
|  | Anatomical labels | MNI coordinates | Size |  |
|  |  | [x, y, z] | [voxels] |  |
|  | 'Middle occipital gyrus (R)' | [33,-86,9] | 141 |  |
|  | 'Middle occipital gyrus (L)' | [-38,-82,12] | 153 |  |
|  | 'Superior occipital gyrus (R)' | [24,-89,20] | 55 |  |
|  | 'Fusiform gyrus (L)' | [-30,-68,-12] | 64 |  |
|  | 'Inferior occipital gyrus (R)' | [41,-81,-6] | 44 |  |
|  | 'Superior occipital gyrus (L)' | [-18,-91,32] | 24 |  |
|  | 'Cuneus (R)' | [18,-96,11] | 15 |  |
|  | 'Fusiform gyrus (R)' | [30,-72,-11] | 20 |  |
|  | 'Inferior occipital gyrus (L)' | [-33,-76,-9] | 10 |  |
|  | 'Calcarine fissure and surrounding cortex (R)' | [7,-88,3] | 14 |  |
|  | 'Lingual gyrus (L)' | [-22,-72,-12] | 13 |  |
|  | 'Lingual gyrus (R)' | [26,-66,-1] | 9 |  |
|  | 'Calcarine fissure and surrounding cortex (L)' | [4,-90,5] | 5 |  |
|  | 'Cuneus (L)' | [-10,-89,37] | 2 |  |
|  |  |  |  |  |

Table 6. Cluster topology interaction between gender judgment and naturalistic listening.

|  |  |  |  |  |
| --- | --- | --- | --- | --- |
|  | i1 (C4_N∩C3_G) |  |  |  |
|  | Anatomical labels | MNI coordinates | Size |  |
|  |  | [x, y, z] | [voxels] |  |
|  | 'Temporal pole, middle temporal gyrus (L)' | [-49,14,-28] | 9 |  |
|  | 'Inferior frontal gyrus, orbital part (R)' | [39,26,-20] | 9 |  |
|  |  |  |  |  |
|  | i2 (C6_N∩C3_G) |  |  |  |
|  | Anatomical labels | MNI coordinates | Size |  |
|  |  | [x, y, z] | [voxels] |  |
|  | 'Temporal pole, superior temporal gyrus (L)' | [-37,10,-23] | 143 |  |
|  | 'Parahippocampal gyrus (L)' | [-16,-9,-23] | 106 |  |
|  | 'Lobule III of cerebellum (R)' | [12,-33,-19] | 46 |  |
|  | 'Temporal pole, superior temporal gyrus (R)' | [40,9,-22] | 68 |  |
|  | 'Lobule III of cerebellum (L)' | [-11,-35,-20] | 30 |  |
|  | 'Parahippocampal gyrus (R)' | [19,-7,-22] | 39 |  |
|  | 'Superior temporal gyrus (L)' | [-46,-6,-9] | 49 |  |
|  | 'Lobules IV-V of cerebellum (L)' | [-13,-35,-18] | 36 |  |
|  | 'Vermis III of cerebellum' | [2,-39,-12] | 16 |  |
|  | 'Amygdala (L)' | [-21,1,-21] | 15 |  |
|  | 'Amygdala (R)' | [29,3,-27] | 6 |  |
|  | 'Insula (R)' | [43,3,-9] | 14 |  |
|  | 'Vermis I-II of cerebellum' | [0,-37,-19] | 3 |  |
|  | 'Hippocampus (L)' | [-14,-6,-21] | 9 |  |
|  | 'Temporal pole, middle temporal gyrus (L)' | [-40,9,-28] | 5 |  |
|  | 'Olfactory cortex (L)' | [-2,10,-14] | 1 |  |
|  |  |  |  |  |
|  | i3 (C3_G∩C9_N) |  |  |  |
|  | Anatomical labels | MNI coordinates | Size |  |
|  |  | [x, y, z] | [voxels] |  |
|  | 'Superior temporal gyrus (L)' | [-52,-9,-1] | 11 |  |
|  | 'Insula (R)' | [43,-11,-2] | 10 |  |
|  |  |  |  |  |
|  | i4 (C6_N∩C5_G) |  |  |  |
|  | Anatomical labels | MNI coordinates | Size |  |
|  |  | [x, y, z] | [voxels] |  |
|  | 'Insula (R)' | [43,3,-8] | 9 |  |
|  | 'Superior temporal gyrus (L)' | [-48,-2,-6] | 2 |  |
|  |  |  |  |  |
|  | i5 (C5_G∩C8_N) |  |  |  |
|  | Anatomical labels | MNI coordinates | Size |  |
|  |  | [x, y, z] | [voxels] |  |
|  | 'Insula (R)' | [39,19,-2] | 4 |  |
|  | 'Inferior frontal gyrus, opercular part (R)' | [52,17,4] | 2 |  |
|  |  |  |  |  |
|  | i6 (C5-G∩C9_N) |  |  |  |
|  | Anatomical labels | MNI coordinates | Size |  |
|  |  | [x, y, z] | [voxels] |  |
|  | 'Superior temporal gyrus (R)' | [55,-18,4] | 50 |  |
|  | 'Superior temporal gyrus (L)' | [-54,-13,2] | 24 |  |
|  | 'Heschl''s gyrus (R)' | [45,-18,7] | 8 |  |
|  | 'Insula (R)' | [45,-7,-1] | 8 |  |
|  | 'Middle temporal gyrus (L)' | [-57,-27,0] | 8 |  |
|  | 'Heschl''s gyrus (L)' | [-50,-16,7] | 2 |  |
|  |  |  |  |  |
|  | C6_N − i2 (C6_N∩C3_G) − i4 (C6_N∩C5_G) | | |  |
|  | Anatomical labels | MNI coordinates | Size |  |
|  |  | [x, y, z] | [voxels] |  |
|  | 'Temporal pole, superior temporal gyrus (R)' | [35,8,-21] | 15 |  |
|  | 'Vermis III of cerebellum' | [-1,-38,-12] | 8 |  |
|  | 'Lobule III of cerebellum (R)' | [12,-35,-18] | 7 |  |
|  | 'Temporal pole, superior temporal gyrus (L)' | [-40,11,-20] | 10 |  |
|  | 'Superior temporal gyrus (L)' | [-45,-6,-9] | 12 |  |
|  | 'Insula (R)' | [41,14,-11] | 10 |  |
|  | 'Lobules IV-V of cerebellum (L)' | [-11,-41,-10] | 8 |  |
|  | 'Lingual gyrus (L)' | [-11,-36,-7] | 7 |  |
|  | 'Vermis I-II of cerebellum' | [1,-37,-19] | 2 |  |
|  | 'Lobule III of cerebellum (L)' | [-4,-38,-16] | 1 |  |
|  | 'Parahippocampal gyrus (R)' | [16,-3,-18] | 2 |  |
|  | 'Parahippocampal gyrus (L)' | [-20,-28,-24] | 1 |  |
|  |  |  |  |  |
|  | C3_G − i1 (C4_N∩C3_G) − i2 (C6_N∩C3_G) − i3 (C3_G∩C9_N) | | |  |
|  | Anatomical labels | MNI coordinates | Size |  |
|  |  | [x, y, z] | [voxels] |  |
|  | 'Temporal pole, superior temporal gyrus (L)' | [-41,13,-22] | 453 |  |
|  | 'Temporal pole, superior temporal gyrus (R)' | [41,13,-23] | 341 |  |
|  | 'Crus I of cerebellum (L)' | [-30,-80,-25] | 374 |  |
|  | 'Crus I of cerebellum (R)' | [40,-76,-28] | 218 |  |
|  | 'Superior temporal gyrus (L)' | [-48,-5,-8] | 196 |  |
|  | 'Parahippocampal gyrus (L)' | [-18,-11,-22] | 128 |  |
|  | 'Temporal pole, middle temporal gyrus (L)' | [-41,14,-31] | 112 |  |
|  | 'Vermis III of cerebellum' | [2,-40,-12] | 61 |  |
|  | 'Lobules IV-V of cerebellum (L)' | [-11,-40,-13] | 113 |  |
|  | 'Parahippocampal gyrus (R)' | [22,-2,-25] | 95 |  |
|  | 'Insula (R)' | [40,4,-9] | 115 |  |
|  | 'Temporal pole, middle temporal gyrus (R)' | [44,14,-29] | 83 |  |
|  | 'Amygdala (L)' | [-22,0,-20] | 46 |  |
|  | 'Vermis IV-V of cerebellum' | [1,-55,-1] | 64 |  |
|  | 'Lobule III of cerebellum (L)' | [-7,-39,-16] | 37 |  |
|  | 'Lobule III of cerebellum (R)' | [12,-35,-19] | 42 |  |
|  | 'Thalamus (L)' | [-3,-21,8] | 66 |  |
|  | 'Hippocampus (L)' | [-19,-19,-15] | 53 |  |
|  | 'Amygdala (R)' | [29,1,-24] | 28 |  |
|  | 'Crus II of cerebellum (L)' | [-8,-87,-26] | 59 |  |
|  | 'Inferior frontal gyrus, orbital part (R)' | [35,22,-17] | 46 |  |
|  | 'Vermis VI of cerebellum' | [-1,-73,-10] | 17 |  |
|  | 'Lobules IV-V of cerebellum (R)' | [10,-44,-9] | 27 |  |
|  | 'Olfactory cortex (L)' | [0,12,-9] | 14 |  |
|  | 'Vermis I-II of cerebellum' | [1,-38,-20] | 5 |  |
|  | 'Gyrus rectus (L)' | [0,28,-18] | 21 |  |
|  | 'Inferior temporal gyrus (L)' | [-56,-55,-24] | 31 |  |
|  | 'Olfactory cortex (R)' | [30,10,-20] | 1 |  |
|  | 'Gyrus rectus (R)' | [1,23,-17] | 4 |  |
|  | 'Hippocampus (R)' | [21,-3,-21] | 3 |  |
|  | 'Caudate nucleus (L)' | [-2,15,0] | 2 |  |
|  | 'Fusiform gyrus (L)' | [-18,-35,-19] | 8 |  |
|  | 'Inferior temporal gyrus (R)' | [54,-55,-26] | 13 |  |
|  |  |  |  |  |
|  | C5_G − i4 (C6_N∩C5_G) − i5 (C5_G∩C8_N) − i6 (C5-G∩C9_N) | | |  |
|  | Anatomical labels | MNI coordinates | Size |  |
|  |  | [x, y, z] | [voxels] |  |
|  | 'Insula (R)' | [40,-2,3] | 394 |  |
|  | 'Superior temporal gyrus (R)' | [55,-24,6] | 612 |  |
|  | 'Superior temporal gyrus (L)' | [-53,-18,4] | 379 |  |
|  | 'Rolandic operculum (R)' | [49,-10,13] | 231 |  |
|  | 'Rolandic operculum (L)' | [-47,-11,10] | 182 |  |
|  | 'Heschl''s gyrus (R)' | [45,-18,9] | 100 |  |
|  | 'Insula (L)' | [-42,0,0] | 158 |  |
|  | 'Heschl''s gyrus (L)' | [-43,-18,9] | 70 |  |
|  | 'Middle temporal gyrus (L)' | [-56,-25,-1] | 225 |  |
|  | 'Postcentral gyrus (R)' | [57,-15,38] | 181 |  |
|  | 'Inferior frontal gyrus, opercular part (R)' | [49,14,11] | 86 |  |
|  | 'Inferior frontal gyrus, opercular part (L) ' | [-55,10,11] | 72 |  |
|  | 'Precentral gyrus (R)' | [58,2,33] | 115 |  |
|  | 'Supramarginal gyrus (R)' | [53,-31,25] | 56 |  |
|  | 'Supplementary motor area (R)' | [9,-3,63] | 30 |  |
|  | 'Putamen (R)' | [33,7,7] | 3 |  |
|  |  |  |  |  |
|  | C9_N − i3 (C3_G∩C9_N) − i6 (C5-G∩C9_N) | | |  |
|  | Anatomical labels | MNI coordinates | Size |  |
|  |  | [x, y, z] | [voxels] |  |
|  | 'Superior temporal gyrus (R)' | [58,-16,2] | 127 |  |
|  | 'Superior temporal gyrus (L)' | [-58,-11,2] | 34 |  |
|  | 'Middle temporal gyrus (L)' | [-60,-27,-2] | 31 |  |
|  | 'Heschl''s gyrus (R)' | [48,-20,7] | 3 |  |
|  | 'Insula (R)' | [43,-7,0] | 6 |  |
|  |  |  |  |  |
|  | C4_N − i1 (C4_N∩C3_G) |  |  |  |
|  | Anatomical labels | MNI coordinates | Size |  |
|  |  | [x, y, z] | [voxels] |  |
|  | 'Temporal pole, superior temporal gyrus (L)' | [-40,12,-22] | 596 |  |
|  | 'Temporal pole, superior temporal gyrus (R)' | [41,12,-23] | 409 |  |
|  | 'Crus I of cerebellum (L)' | [-30,-80,-25] | 374 |  |
|  | 'Parahippocampal gyrus (L)' | [-17,-10,-22] | 234 |  |
|  | 'Superior temporal gyrus (L)' | [-48,-5,-8] | 256 |  |
|  | 'Crus I of cerebellum (R)' | [40,-76,-28] | 218 |  |
|  | 'Lobule III of cerebellum (R)' | [12,-34,-19] | 88 |  |
|  | 'Lobules IV-V of cerebellum (L)' | [-12,-39,-14] | 149 |  |
|  | 'Vermis III of cerebellum' | [2,-40,-12] | 77 |  |
|  | 'Lobule III of cerebellum (L)' | [-9,-37,-18] | 67 |  |
|  | 'Temporal pole, middle temporal gyrus (L)' | [-41,14,-31] | 117 |  |
|  | 'Parahippocampal gyrus (R)' | [21,-3,-24] | 134 |  |
|  | 'Amygdala (L)' | [-22,0,-20] | 61 |  |
|  | 'Insula (R)' | [40,3,-8] | 139 |  |
|  | 'Temporal pole, middle temporal gyrus (R)' | [44,14,-29] | 83 |  |
|  | 'Vermis IV-V of cerebellum' | [1,-55,-1] | 64 |  |
|  | 'Amygdala (R)' | [29,1,-24] | 34 |  |
|  | 'Hippocampus (L)' | [-19,-17,-16] | 62 |  |
|  | 'Thalamus (L)' | [-3,-21,8] | 66 |  |
|  | 'Crus II of cerebellum (L)' | [-8,-87,-26] | 59 |  |
|  | 'Vermis I-II of cerebellum' | [1,-38,-20] | 8 |  |
|  | 'Inferior frontal gyrus, orbital part (R)' | [35,22,-17] | 46 |  |
|  | 'Olfactory cortex (L)' | [0,12,-9] | 15 |  |
|  | 'Vermis VI of cerebellum' | [-1,-73,-10] | 17 |  |
|  | 'Lobules IV-V of cerebellum (R)' | [10,-44,-9] | 27 |  |
|  | 'Gyrus rectus (L)' | [0,28,-18] | 21 |  |
|  | 'Olfactory cortex (R)' | [30,10,-20] | 1 |  |
|  | 'Inferior temporal gyrus (L)' | [-56,-55,-24] | 31 |  |
|  | 'Gyrus rectus (R)' | [1,23,-17] | 4 |  |
|  | 'Hippocampus (R)' | [21,-3,-21] | 3 |  |
|  | 'Caudate nucleus (L)' | [-2,15,0] | 2 |  |
|  | 'Fusiform gyrus (L)' | [-18,-35,-19] | 8 |  |
|  | 'Inferior temporal gyrus (R)' | [54,-55,-26] | 13 |  |
|  |  |  |  |  |
|  | C8_N − i5 (C5_G∩C8_N) |  |  |  |
|  | Anatomical labels | MNI coordinates | Size |  |
|  |  | [x, y, z] | [voxels] |  |
|  | 'Insula (R)' | [41,-2,3] | 411 |  |
|  | 'Superior temporal gyrus (R)' | [55,-24,6] | 662 |  |
|  | 'Superior temporal gyrus (L)' | [-53,-18,4] | 405 |  |
|  | 'Rolandic operculum (R)' | [49,-10,13] | 231 |  |
|  | 'Rolandic operculum (L)' | [-47,-11,10] | 182 |  |
|  | 'Heschl''s gyrus (R)' | [45,-18,9] | 108 |  |
|  | 'Heschl''s gyrus (L)' | [-43,-18,9] | 72 |  |
|  | 'Insula (L)' | [-42,0,0] | 158 |  |
|  | 'Middle temporal gyrus (L)' | [-56,-25,-1] | 233 |  |
|  | 'Postcentral gyrus (R)' | [57,-15,38] | 181 |  |
|  | 'Inferior frontal gyrus, opercular part (R)' | [49,14,11] | 86 |  |
|  | 'Inferior frontal gyrus, opercular part (L) ' | [-55,10,11] | 72 |  |
|  | 'Precentral gyrus (R)' | [58,2,33] | 115 |  |
|  | 'Supramarginal gyrus (R)' | [53,-31,25] | 56 |  |
|  | 'Supplementary motor area (R)' | [9,-3,63] | 30 |  |
|  | 'Putamen (R)' | [33,7,7] | 3 |  |
|  |  |  |  |  |
